# Anti-tumor immunity enhancement by photodynamic therapy with talaporfin sodium and anti-programmed death 1 antibody

**DOI:** 10.1016/j.omto.2022.12.009

**Published:** 2023-01-02

**Authors:** Makiko Sasaki, Mamoru Tanaka, Yuki Kojima, Hirotada Nishie, Takaya Shimura, Eiji Kubota, Hiromi Kataoka

**Affiliations:** 1 Department of Gastroenterology and Metabolism, Nagoya City University Graduate School of Medical Science, 1 Kawasumi, Mizuho-cho, Mizuho-ku, Nagoya, Aichi 467-8601, Japan

**Keywords:** photodynamic therapy, talaporfin sodium, immunogenic cell death, immune checkpoint inhibitor, anti-PD-1 antibody

## Abstract

Photodynamic therapy (PDT) is a relatively non-invasive anti-cancer therapy that employs a photosensitizer with a specific wavelength of light irradiation. PDT induces direct cell killing and enhancement effects on tumor immunity, but its underlying mechanism remains unknown. Here, we perform a basic analysis of the anti-tumor effect of talaporfin sodium (TS)-PDT as well as its synergism with the immune checkpoint inhibitor anti-programmed death 1 (anti-PD-1) antibody. We estimate the cell death mechanism induced by TS-PDT and the induction of damage-associated molecular patterns (DAMPs) by TS-PDT *in vitro*. We establish a syngeneic mouse model of bilateral flank tumors and verify the enhancement of the abscopal effect on the non-irradiated side. TS-PDT induced apoptosis, necrosis, and autophagy-associated cell death *in vitro*. TS-PDT induced the release and/or expression of DAMPs *in vitro*. Tumor growth was inhibited in the TS-PDT and anti-PD-1 antibody combination group compared with other single-treatment or non-treatment groups *in vivo*. In summary, TS-PDT induces the release and/or expression of DAMPs, indicating that it activates innate immunity. PD-1 blockage enhances the anti-tumor immunity induced by TS-PDT. Thus, our results demonstrate that the combination of TS-PDT and anti-PD-1 antibody can potentially be used for anti-tumor therapy.

## Graphical abstract

**Figure:**
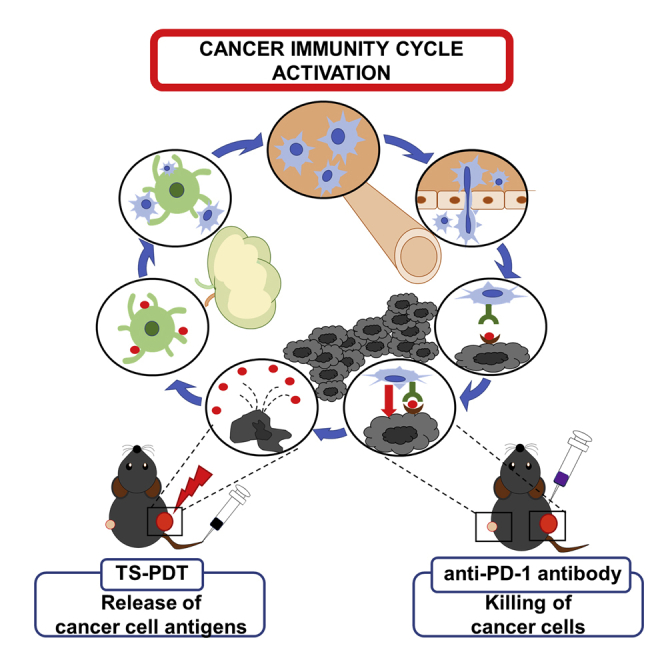

## Teaser

Photodynamic therapy is a relatively non-invasive anti-cancer therapy, and anti-tumor immunity enhancement by photodynamic therapy with talaporfin sodium and anti-programmed death 1 antibody would open the way to a bright future for cancer therapy.

## Introduction

The development of non-invasive treatment methods for cancers is desired for the aging population. From this perspective, photodynamic therapy (PDT), an anti-cancer therapy that uses photosensitizers (PSs) with specific wavelengths of light irradiation,^1^ has attracted a great deal of attention. It is relatively non-invasive, as irradiation is limited to the cancer site, and the PS predominantly accumulates in the tumor cells, thereby showing less systemic toxicity. Tumor destruction by PDT is a multifactorial process that involves: (1) direct killing of tumor cells by inducing reactive oxygen species, (2) tumor vessel damage, and (3) induction of anti-cancer immunity via the activation of cytotoxic T lymphocytes (CTLs).^2^^,^^3^^,^^4^ First-generation PDT using porfimer sodium has some disadvantages, such as skin phototoxicity, a long sunshade period requirement, and the need for an expensive and large laser system for excitation.^5^ However, second-generation PDT using talaporfin sodium (TS) has overcome the disadvantages of the first-generation PDT.^6^^,^^7^ In this study, we focused on the anti-cancer adaptive immunity induced by TS-PDT.

Radiotherapy at one site occasionally leads to the regression of metastatic tumors that are not irradiated. This phenomenon was named the “abscopal effect.”^8^^,^^9^ The abscopal effect has been reported for several cancers,^8^^,^^10^^,^^11^^,^^12^ as well as in the field of PDT.^13^ The abscopal effect has been suggested to involve the immune system, but the mechanism of action has been unsolved. Some studies on the synergistic effect of the abscopal effect using immune checkpoint inhibitors (ICIs) have already been reported in the radiotherapy and PDT fields.^8^^,^^14^^,^^15^^,^^16^ Immunogenicity is enhanced in neoantigen-presenting cells, and induction of CTLs by specific immunity is associated with a tumor-killing effect. PDT induces not only a direct cell-killing effect but also an enhancement effect on tumor immunity,^17^^,^^18^ but its mechanism remains unsolved. Immunogenic cell death (ICD) caused by PDT induces damage-associated molecular patterns (DAMPs),^19^^,^^20^^,^^21^^,^^22^ which activate innate immunity, in turn activating adaptive immunity.^23^^,^^24^ For an effective anti-cancer immune response, a series of stepwise events must be initiated and allowed to proceed and expand iteratively.^25^ This cycle is well known as the “cancer immunity cycle.” In this cycle, many factors can help to drive or suppress anti-cancer immunity at each step. Immunotherapy with programmed death 1 (PD-1)/programmed death ligand 1 (PD-L1) pathway blockage has had a great impact on cancer therapy.^26^^,^^27^^,^^28^ It has emerged that inciting a cancer cell death routine, associated with the activation of danger signaling pathways that induce the emission of DAMPs, markedly increases the immunogenicity of dying cancer cells. The major DAMPs in ICD are calreticulin (CRT), heat-shock protein (HSP), ATP, and high-mobility group protein B1 (HMGB1).^20^

For the above reasons, we hypothesized that the addition of ICIs to PDT regulates the immune system and promotes the cancer immunity cycle, leading to the effective inhibition of tumor growth at both the irradiated and the non-irradiated sites. Here, we performed a basic analysis of the anti-tumor effect of TS-PDT as well as its synergism with the anti-PD-1 antibody.

## Results

### TS accumulated in cancer cells and was mainly localized in the lysosomes

**Figure 1:**
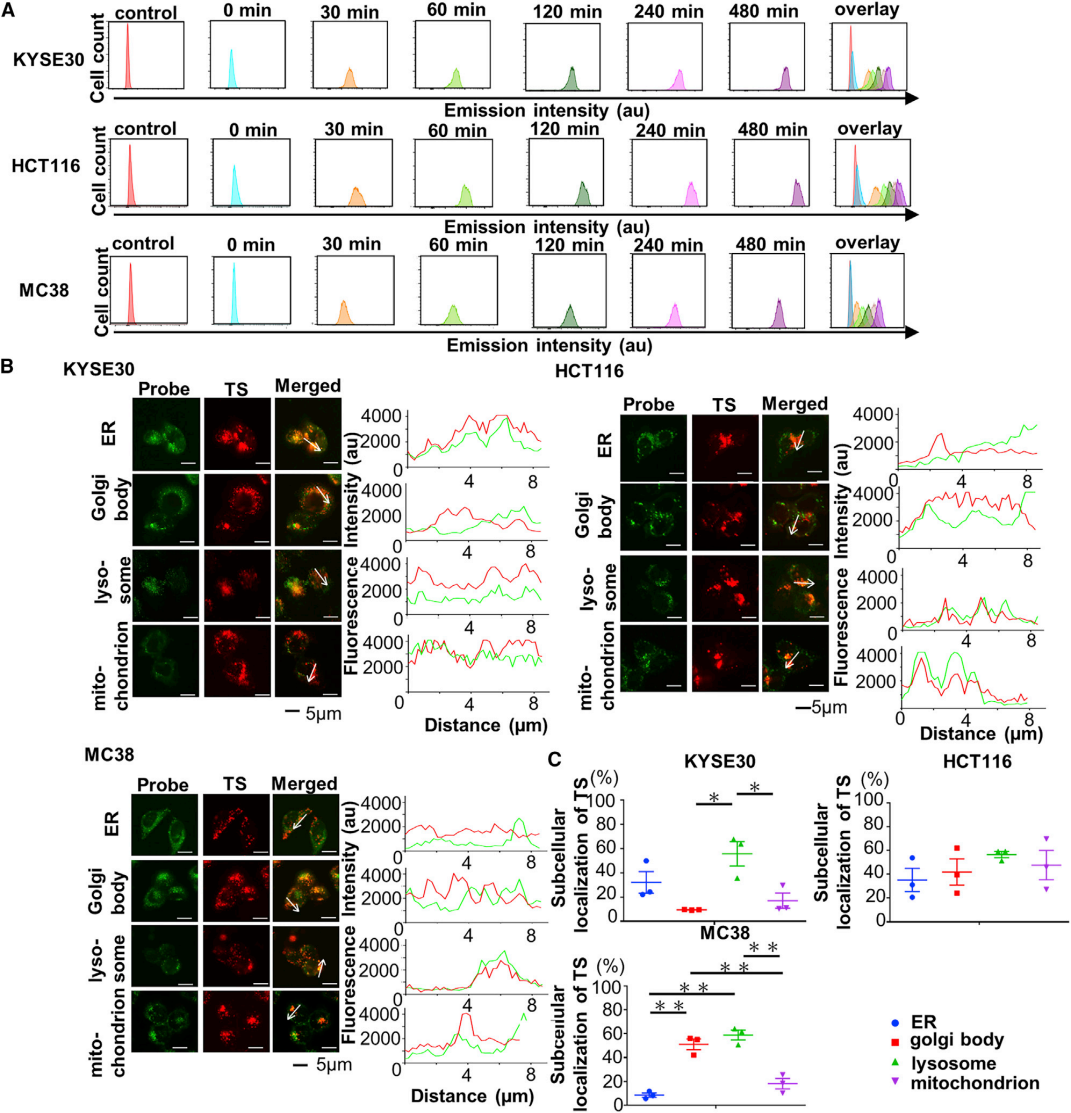
Accumulation and subcellular localization of talaporfin sodium (TS) in cancer cells *in vitro* (A) Histogram data from flow cytometric analysis. The abscissa indicates the intensity of emission and the ordinate represents the number of cells. (B) Subcellular localization of TS. Cells were loaded with TS and labeled with organelle-specific probes. Images were obtained by confocal microscopy (original magnification, ×300; scale bar, 5 μm). Each row represents the fluorescence of organelle-specific probes and TS. The fluorescence intensity profiles of TS (red lines) and organelle probes (green lines) were examined along the arrows in the confocal images. (C) Quantitative analysis of subcellular localization of TS. Data from three independent experiments are presented as the mean ± standard error (SE). Statistical significance was determined using Holm-Sidak’s multiple comparisons test and was set at ∗p < 0.05 and ∗∗p < 0.01.

The accumulation of TS in all three cell lines (KYSE30, HCT116, and MC38) increased in a time-dependent manner (Figure 1A). Next, we examined the subcellular localization of TS by confocal microscopy. The fluorescence intensity profiles for TS detection exhibited a tendency to correlate with the lysosome tracking marker in all three cell lines Figure 1B). The quantitative analysis was performed using the whole image, and the average values were calculated, and the results were almost consistent with the fluorescence intensity profile analysis (Figure 1C).

### TS-PDT induced necrosis, apoptosis, and autophagy-associated cell death

**Figure 2:**
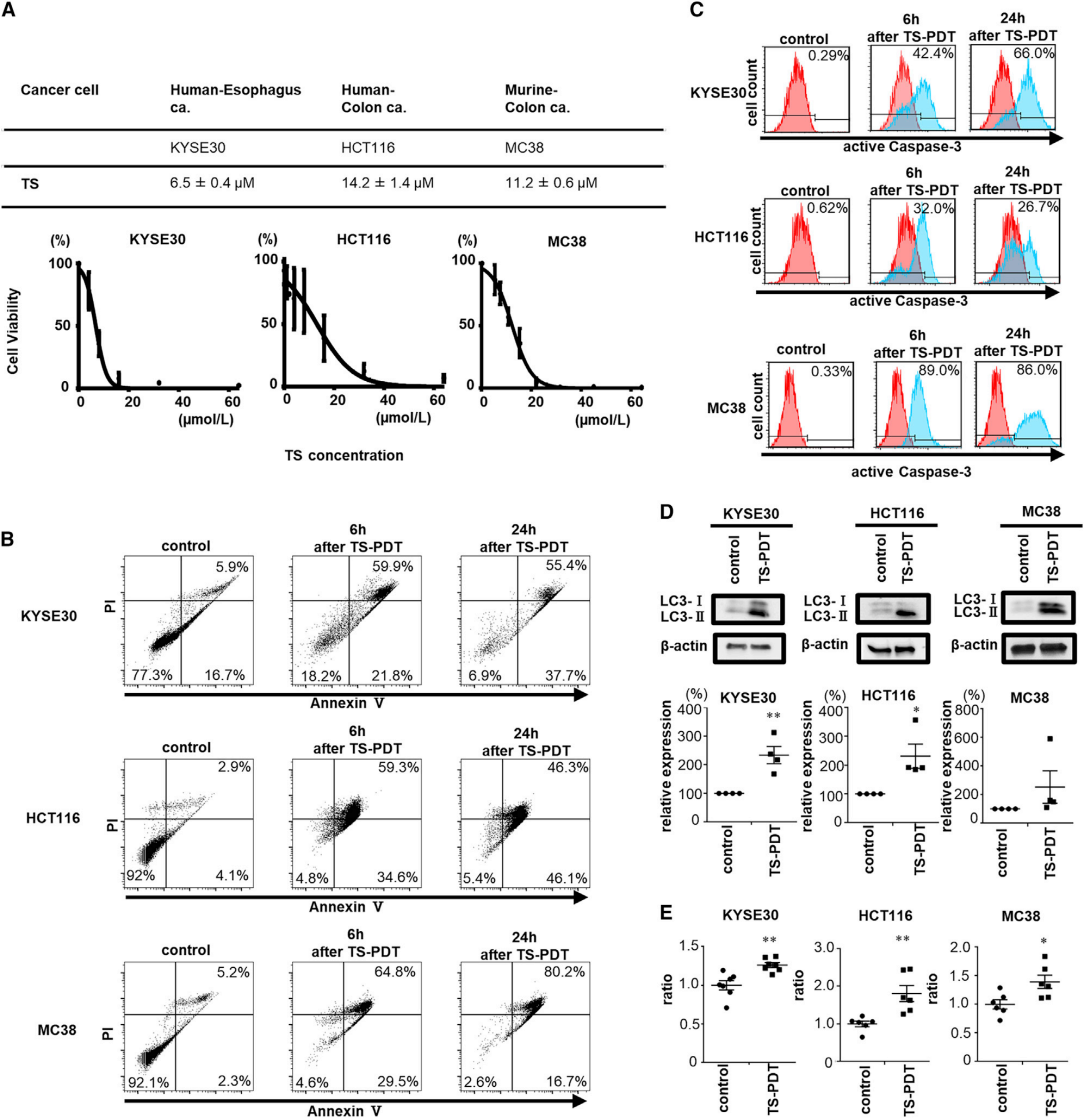
Induction of cell death by TS-photodynamic therapy (TS-PDT) *in vitro* (A) Proliferation assay of cells treated with TS-PDT. Data are presented as the mean ± SE of three independent experiments. (B) Analysis of necrosis/apoptosis using annexin V and propidium iodide (PI) staining. Four populations are indicated as non-apoptotic dead cells (upper left), late apoptosis/necrosis cells (upper right), viable cells (lower left), and early apoptotic cells (lower right). (C) Apoptosis assay for measuring active caspase-3 levels. The cell population is indicated as a histogram. (D) Immunoblotting analysis of LC3. The relative expression of LC3 normalized to β-actin is shown as a bar graph. Statistical significance was determined using Student’s t test and was set at ∗p < 0.05 and ∗∗p < 0.01 (n = 4). (E) Quantitative analysis using the fluorescent dye DAP green to detect autophagosomes and autolysosomes. Statistical significance was determined using Student’s t test and was set at ∗p < 0.05 and ∗∗p < 0.01(n = 6–7).

The WST-8 assay was used to determine the IC_50_ at 24 h after TS-PDT. TS-PDT induced cell death in a dose-dependent manner in all three cell lines (Figure 2A). IC_50_ of 6.5 (±0.4), 14.2 (±1.4), and 11.2 (±0.6) μmol/L for KYSE30, HCT116, and MC38, respectively, was observed. To analyze the cell death mechanism induced by TS-PDT, we performed the apoptosis/necrosis assay and stained with annexin V-fluorescein isothiocyanate (FITC) and propidium iodide (PI) for flow cytometric analysis. The percentage of cells undergoing apoptosis and necrosis was elevated in TS-PDT-treated cells compared with untreated cells (Figure 2B). The mean fluorescence intensity of active caspase-3, a marker of apoptosis, was higher in cells treated with TS-PDT than in untreated cells (Figure 2C). Immunoblot analysis was conducted to assess the expression of the autophagy-related protein LC3. The results showed that the levels of LC3, especially LC3-II, increased after TS-PDT (Figures 2D and S1). To confirm the induction of autophagy-related cell death by TS-PDT, we used DAP green fluorescent dye, which is incorporated into autophagosomal membranes for quantitative analysis. Stronger fluorescence was observed in the TS-PDT-treated groups compared with the untreated group (Figure 2E).

### TS-PDT induced the release and/or expression of DAMPs *in vitro*

**Figure 3:**
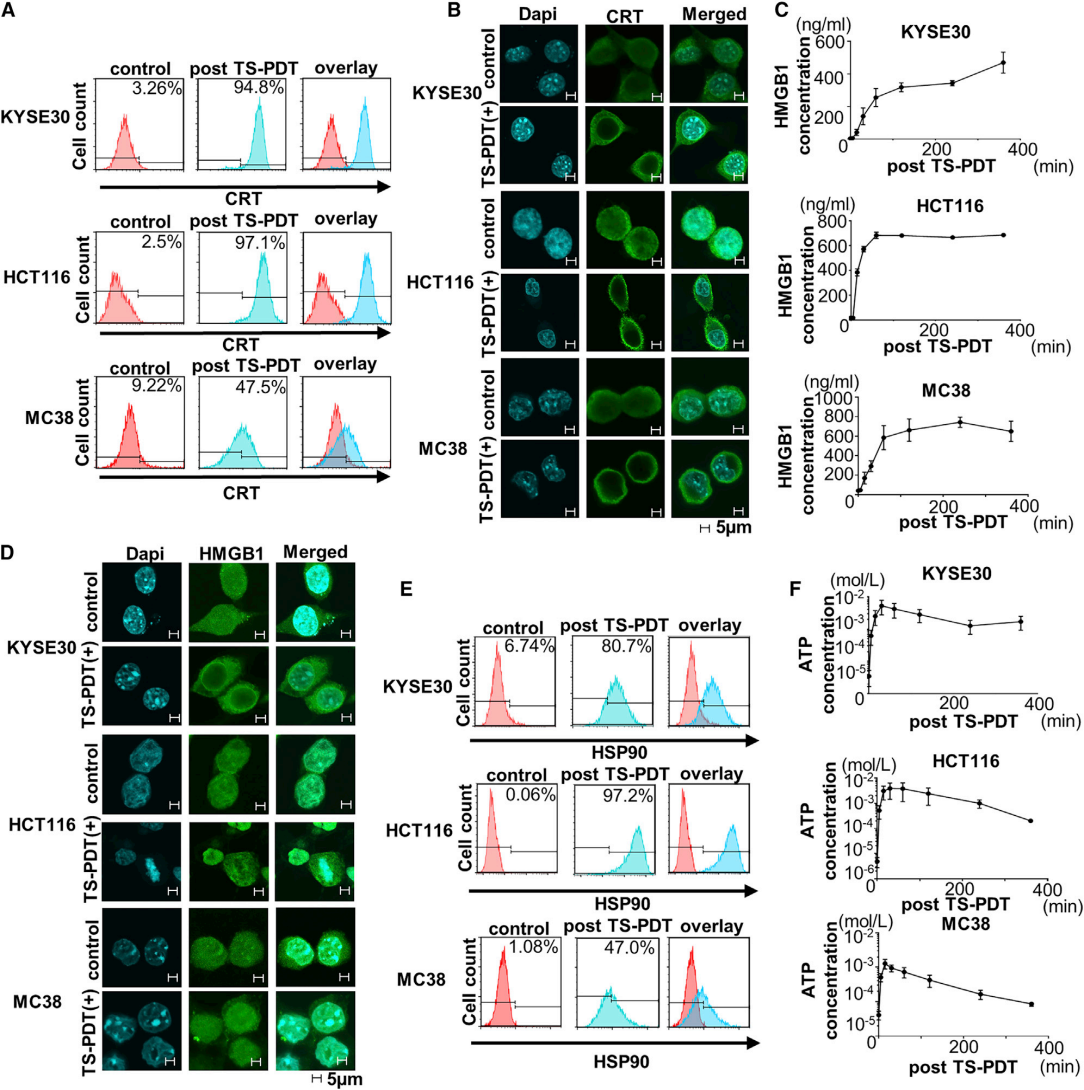
Induction of damage-associated molecular patterns (DAMPs) by TS-PDT *in vitro* (A) Flow cytometric analysis of cell-surface calreticulin (CRT). Negative controls, without TS addition or irradiation, are shown in red. The treated groups are shown in blue. (B) CRT translocation from the endoplasmic reticulum (ER) to the cell surface assessed by immunofluorescence staining. Images were obtained using a confocal microscope (original magnification ×350; scale bar, 5 μm). (C) Extracellular release of high-mobility group protein B1 (HMGB1) induced by TS-PDT assessed by the enzyme-linked immunosorbent assay (ELISA) (n = 3). (D) HMGB1 translocation from the ER to the cell surface was assessed by immunofluorescence staining. Images were obtained using a confocal microscope (original magnification ×350; scale bar, 5 μm). (E) Heat-shock protein (HSP)-90 expression on the cell surface induced by TS-PDT assessed by flow cytometry. Negative controls, without TS addition nor irradiation, are shown in red. The treated groups are shown in blue. (F) Extracellular release of ATP induced by TS-PDT assessed by ELISA. ATP concentration in the culture medium after TS-PDT was detected by ELISA (n = 3).

The TS-PDT-treated cells showed an increase in CRT expression in the plasma membrane (Figure 3A). Furthermore, we measured the translocation of CRT after TS-PDT using immunofluorescence staining. Translocation of CRT to the cytoplasm from the nucleus was induced by TS-PDT (Figure 3B). We measured the supernatant concentration of HMGB1 culture medium treated with TS-PDT to evaluate the release of HMGB1. The concentration of HMGB1 in the supernatants increased after TS-PDT (Figure 3C). Furthermore, we observed the translocation of HMGB1 in cells treated with TS-PDT by immunofluorescence staining (Figure 3D). HMGB1 staining overlapped with DAPI as HMGB1 exited the nucleus in the untreated cells, but after treatment with TS-PDT, HMGB1 tended to be released extracellularly from the nucleus through the permeabilized plasma membrane. The cell-surface exposure of HSP90 was measured by flow cytometry. The increase in HSP90 expression on the cell surface was induced in cells treated with TS-PDT (Figure 3E). The extracellular release of ATP was measured in the supernatant of ATP culture medium treated with TS-PDT. The concentration of ATP in the supernatants rapidly increased after TS-PDT (Figure 3F).

### Anti-PD-1 antibody potentiated the anti-tumor effect of TS-PDT *in vitro*

**Figure 4:**
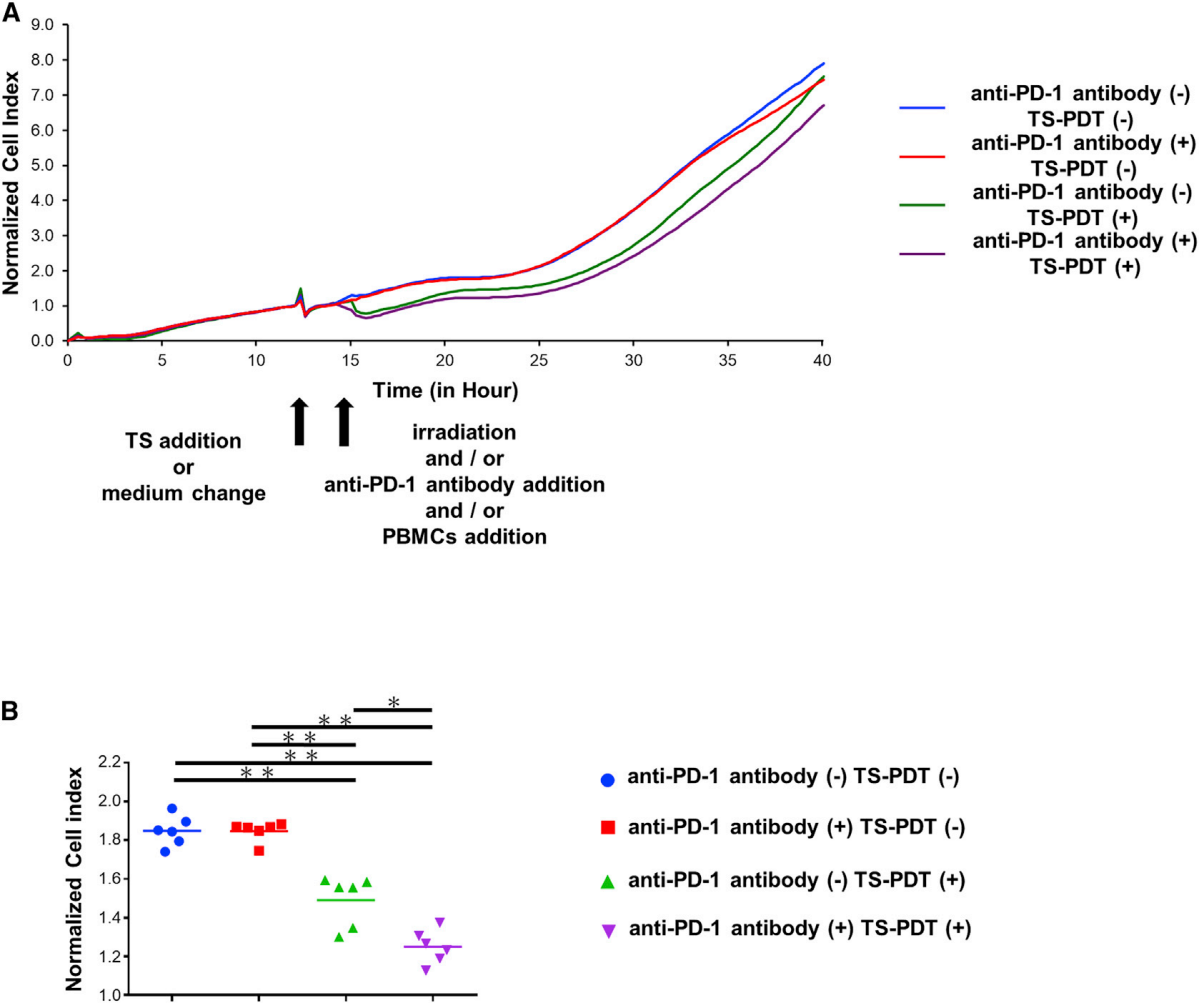
Synergism of TS-PDT with anti-programmed death 1 (anti-PD-1) antibody assessed by the xCELLigence killing assay *in vitro* (A) HCT116 cells were seeded in E-plates and treated with each assigned therapy. The cell index reflects cell viability and was normalized at the time of therapeutic intervention. (B) Same data as from (A) but compared with each group at one specific time point (23:03:42) shown as a scatterplot with error bars. Statistical significance was determined using Holm-Sidak’s multiple comparisons test and was set at ∗p < 0.05 and ∗∗p < 0.01 (n = 6).

To estimate the complex interaction of anti-cancer immunity and efficacy of TS-PDT, a real-time cytolytic *in vitro* potency assay was performed. As shown in Figure 4, a low concentration of anti-PD-1 antibody (10 nM) did not show a cell-killing effect compared with only staphylococcal enterotoxin B (SEB)-stimulated peripheral blood mononuclear cells (PBMCs), and TS-PDT using a low dose of TS (4 μM) led to a slight cell-killing effect. However, the cell-killing effect was increased by a combination of a low dose of anti-PD-1 antibody and TS-PDT (Figure 4). The combination of TS-PDT and anti-PD-1 antibody induced a stronger cell-killing effect compared with TS-PDT alone (p < 0.05) (combination index = 0.31). Preliminary data from this experiment are in Figure S2.

### Anti-PD-1 antibody potentiated the anti-tumor effect of TS-PDT in syngeneic immunocompetent mouse tumor model

**Figure 5:**
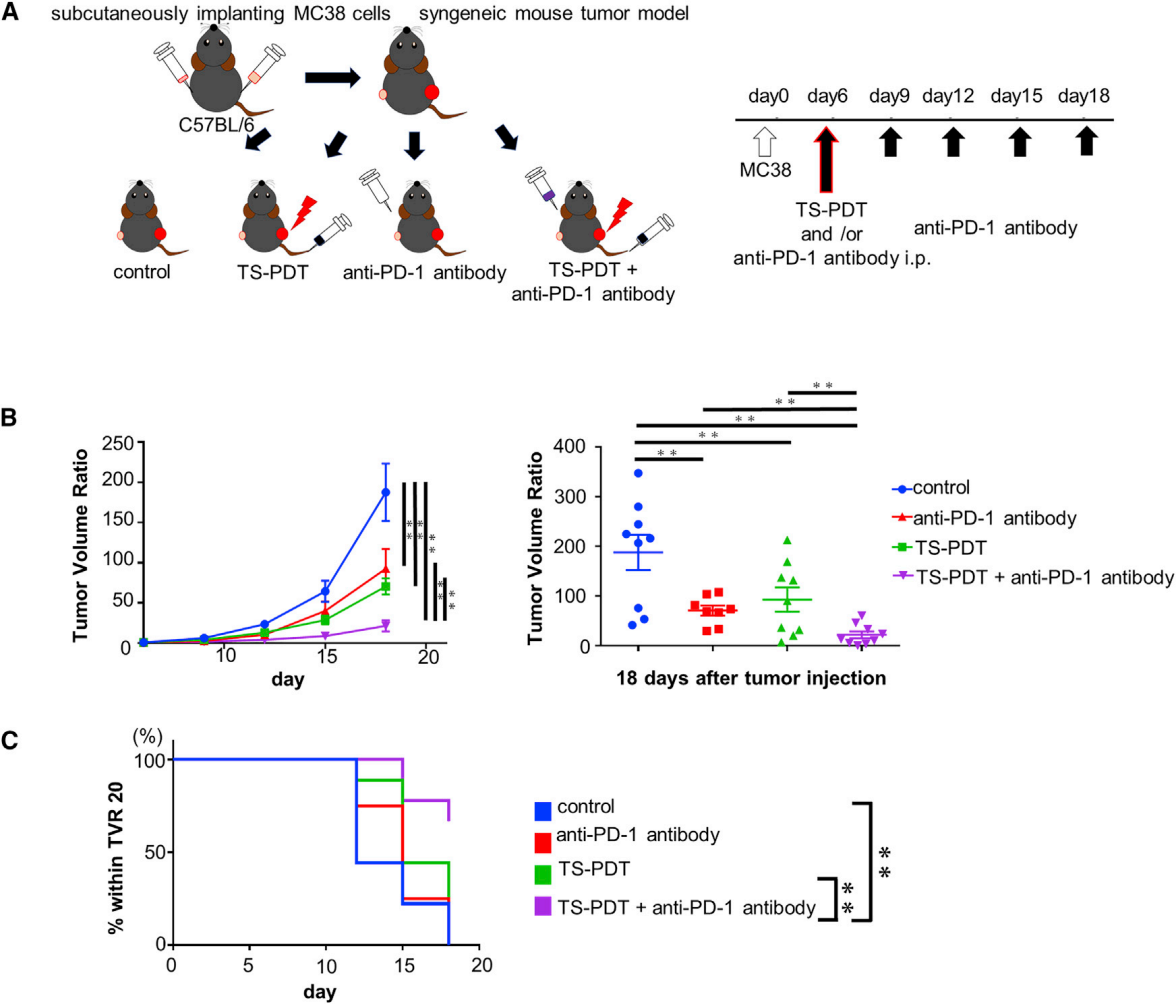
Tumor volume suppression in the non-irradiated side induced by synergism of TS-PDT with anti-PD-1 antibody *in vivo* (A) The murine syngeneic immunocompetent model and experimental design. (B) Tumor volume ratio (TVR) plotted against the number of days and on day 18 (endpoint) after tumor implantation. Values are expressed as the mean ± SE (n = 8–9). ∗∗p < 0.01 (two-way ANOVA with Holm-Sidak’s multiple comparisons test). (C) Percentage within TVR 20 curves constructed by Kaplan-Meier analysis. ∗∗p < 0.01 (log-rank Mantel-Cox test).

We compared the synergism of combination therapy with TS-PDT alone or anti-PD-1 antibody alone through the suppression of tumor growth in each group (Figure 5A). In an MC38 syngeneic immunocompetent mouse tumor model, tumor growth on the irradiated side was suppressed in the TS-PDT group and the anti-PD-1 antibody group compared with the control (untreated) group. Moreover, the TS-PDT and anti-PD-1 antibody combination therapy group showed a significant decrease compared with the tumor volume seen in the monotherapy groups (Figure S3). On the other hand, tumor growth on the non-irradiated side was suppressed not only in the anti-PD-1 antibody group but also in the TS-PDT group, compared with the control group, which was considered to be induced by the abscopal effect. The combination therapy of TS-PDT and anti-PD-1 antibody showed a significant decrease in tumor volume compared with the monotherapy group (Figures 5B and 5C). Thus, this result suggests that anti-PD-1 antibody treatment enhances the abscopal effect induced by TS-PDT.

### TS-PDT induced cytotoxic T lymphocytes and the effect was enhanced by combination with the anti-PD-1 antibody *in vivo*

**Figure 6:**
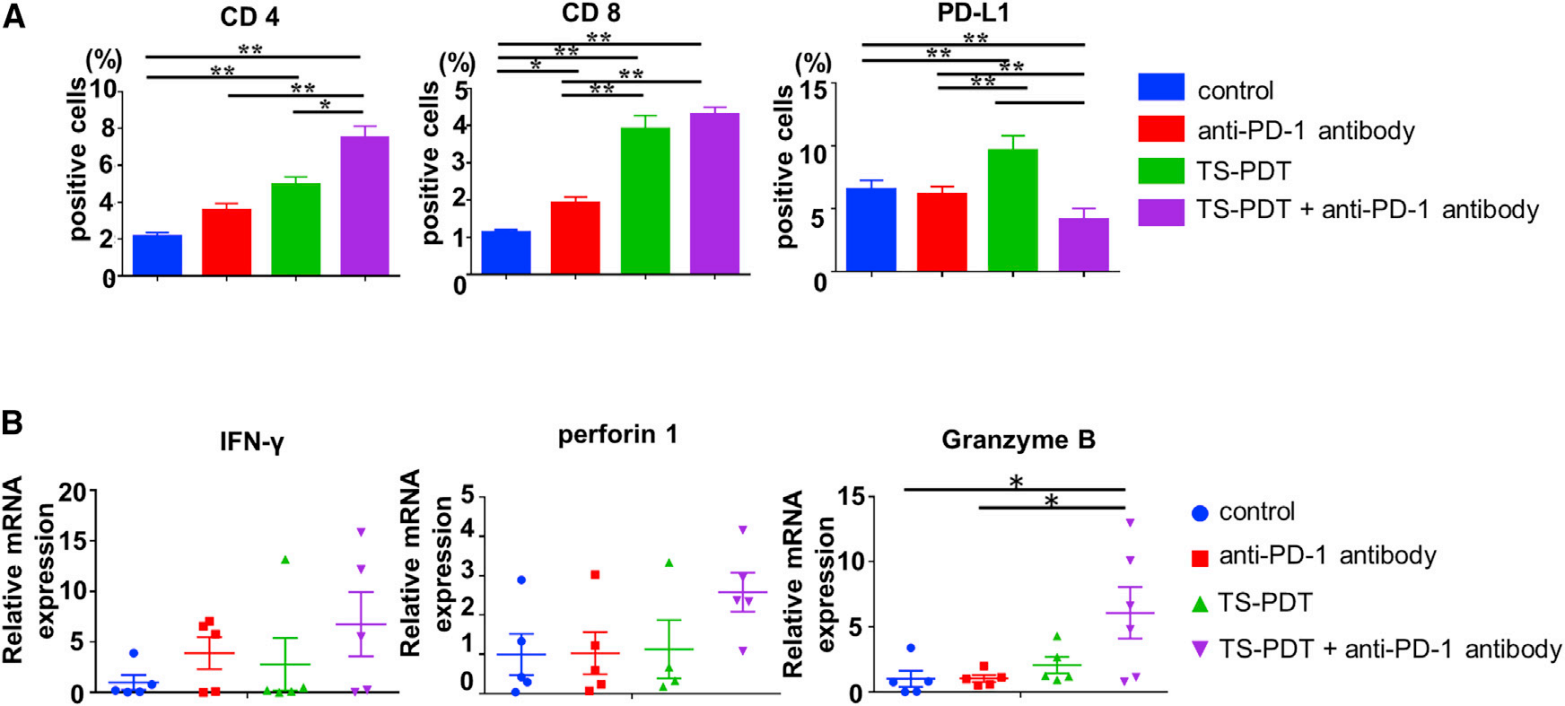
Enhancement of cytotoxic T lymphocyte induction by the combination of TS-PDT and anti-PD-1 antibody *in vivo* (A) Expression levels of cluster of differentiation (CD) 4, CD8, and PD-L1 in non-irradiated side tumors. CD4, CD8, and PD-L1 expression levels were evaluated using immunohistochemistry. Values are expressed as the mean ± SE (n = 5 samples of each group × 15 random fields). ∗p < 0.05 and ∗∗p < 0.01 (Holm-Sidak’s multiple comparisons test). (B) Expression levels of effector molecules released from CD8^+^ effector T cells in non-irradiated side tumors. IFN-γ, perforin 1, and granzyme B expression levels were evaluated by quantitative reverse transcription PCR and normalized against that of glyceraldehyde 3-phosphate dehydrogenase. Values are expressed as the mean ± SE (n = 5). ∗p < 0.05 (Holm-Sidak’s multiple comparisons test).

For immunohistochemical analysis, we examined the expression levels of cluster of differentiation 4 (CD4), CD8, and PD-L1. In the tumors on the non-irradiated sides, the expression levels of CD4, CD8, and PD-L1 were elevated in the TS-PDT-treated group. The combination of TS-PDT and anti-PD-1 antibody reduced PD-L1 expression (Figures 6A and S4A). This result indicates that TS-PDT induces anti-tumor effects not only on irradiated sites but also on distant metastases that are not irradiated directly. Moreover, the addition of anti-PD-1 antibody reduced the expression of PD-L1, which was enhanced by TS-PDT. In the tumors on the non-irradiated side, combination therapy tended to induce IFN-γ, perforin 1, and granzyme B (Figure 6B). The analysis of the irradiated side is shown in Figures S4B and S4C. As for proof of the induction of CTLs, we examined the expression levels of DAMPs (CRT and HMGB1). DAMPs tended to be induced in the TS-PDT and anti-PD-1 antibody-treated group rather than the untreated group or monotherapy group. This result suggested that TS-PDT induced CTLs followed by the release of DAMPs. Moreover, this trend was enhanced by the addition of anti-PD-1 antibody (Figure S4D).

## Discussion

ICD is a functionally peculiar form of regulated cell death that activates an adaptive immune response specific for endogenous or exogenous antigens expressed by dying cells.^29^^,^^30^^,^^31^ In cancer therapy, studies have reported the relevance of neoantigens in the recognition of cancer cells by intrinsic T cells,^32^ but only certain treatments have been reported to stimulate ICD in anti-cancer therapy.^33^ ICD inducers lead to apoptosis of the target cells and release/exposure of DAMPs from the cells that the ICD inducer worked on.^34^ Using a chemical component that acts as a PS, PDT is considered a physicochemical, rather than an exclusively physical, anti-tumor modality.^33^ Unlike chemotherapy, very little is known about the molecular mechanisms that contribute to the immunostimulatory functions of PDT. TS has been widely used in clinical applications. There are already several reports about PDT-triggered ICD plus ICIs (Table S1),^16^^,^^35^^,^^36^^,^^37^^,^^38^^,^^39^^,^^40^^,^^41^^,^^42^^,^^43^^,^^44^^,^^45^^,^^46^^,^^47^^,^^48^^,^^49^^,^^50^^,^^51^ but the combination of TS-PDT and anti-PD-1 antibody was not examined. So, our study is the first report of the combination of TS-PDT and anti-PD-1 antibody, and that has significance and uniqueness. In this study, we investigated the potential for enhancement of cancer immunity by PDT using TS and elucidated the molecular mechanisms *in vitro* and *in vivo*.

In the latest findings on the cell death mechanisms of PDT, cell death pathways associated with PDT include necrosis, apoptosis, autophagy, and others.^52^ We examined three cell lines, human esophageal squamous cell cancer cell line KYSE30, human colon cancer cell line HCT116, and murine colon cancer cell line MC38. In Japan, TS-PDT has been approved for health insurance coverage in cases of esophageal cancer. An esophageal cancer cell line derived from mouse has not been established for research use. We selected murine colon cancer cell line MC38, which is known as a PD-L1-expressing cell line,^53^ for *in vivo* study. In addition, we examined a human esophageal cell line and colon cancer cell line *in vitro* for association with the *in vivo* study. The accumulation of TS increased in a time-dependent manner. It is reported that TS accumulates in lysosomes in the prostate cancer cell line PC3,^54^ and our result that TS accumulated in lysosomes in all cell lines that we observed was consistent with this previous report.

Following that, we considered the cell death mechanisms induced by TS-PDT. TS-PDT induced apoptosis and necrosis, as previously reported.^55^ Furthermore, we focused on lysosomal damage caused by the subcellular localization of TS. Recent data indicate that PDT-induced cell death and efficacy depend on the specific intracellular location of PS.^56^^,^^57^ Lysosomal damage induces autophagy-associated cell death with higher photodynamic efficiency.^56^^,^^58^^,^^59^ In this study, we assessed the expression of the autophagy-related protein LC3. A lipidated form of LC3 has been shown to be an autophagosomal marker and has been used to study autophagy.^60^ The levels of LC3 increased after TS-PDT, indicating that TS-PDT induced not only apoptosis and necrosis but also autophagy. However, several molecular targets are supposed to be damaged simultaneously, because PS does not accumulate in a single organelle, as our experimental results suggested. Therefore, TS-PDT can induce apoptosis, necrosis, and autophagy-associated cell death *in vitro*.

DAMPs are essential for ICD in cancer cells. It was reported that the emission of DAMPs was initially connected with necrosis that occurred as a result of physicochemical injury causing cell death.^22^ However, the many studies have revealed that necrosis as well as other types of cell death, such as apoptosis, pyroptosis, ferroptosis and NETosis, results in the release/exposure of DAMPs.^61^ We confirmed that several DAMPs, such as CRT, HMGB1, HSP90, and ATP, were induced by TS-PDT in this study. However, the extracellular release of ATP increased rapidly after TS-PDT and decreased over time. It is reported that ATP is a time-resolved DAMP.^62^ Nucleotide signaling is intrinsically short-lived in the extracellular compartment. We attribute these results to the reason that ATP has a shorter lifespan than the other DAMPs. The release mechanisms of the major DAMPs have been reported.^61^^,^^62^ These previous reports support our hypothesis that TS-PDT is an ideal ICD inducer through the induction of various DAMPs. ICD inducers cause apoptosis and release/exposure of DAMPs, and then the DAMPs work as either adjuvant or danger signals for the immune system. Following this, the cells that escape direct cell death are finally induced to die by stimulation of the recruitment of dendritic cells (DCs) into the tumor and activation of T cells.

It has been reported that the combination of PD-1/PD-L1 pathway blockade and local radiotherapy could lead to the systemic control of tumors that are refractory to treatment with PD-1/PD-L1pathway blockade alone.^63^ Blockade of PD-1, an inhibitory receptor expressed by T cells, can overcome immune resistance. In our *in vitro* study, the combination of TS-PDT and anti-PD-1 antibody induced a stronger cell-killing effect compared with TS-PDT alone (p < 0.05). Moreover, the combination therapy of TS-PDT and anti-PD-1 antibody had a synergistic effect, which was analyzed by a combination index. This experimental design had a minor limitation in that the host was different in effector cells (PBMCs) and target cells (HCT116). To overcome this limitation, PBMCs were treated with SEB to trigger polyclonal T cell activation.^64^

**Figure 7:**
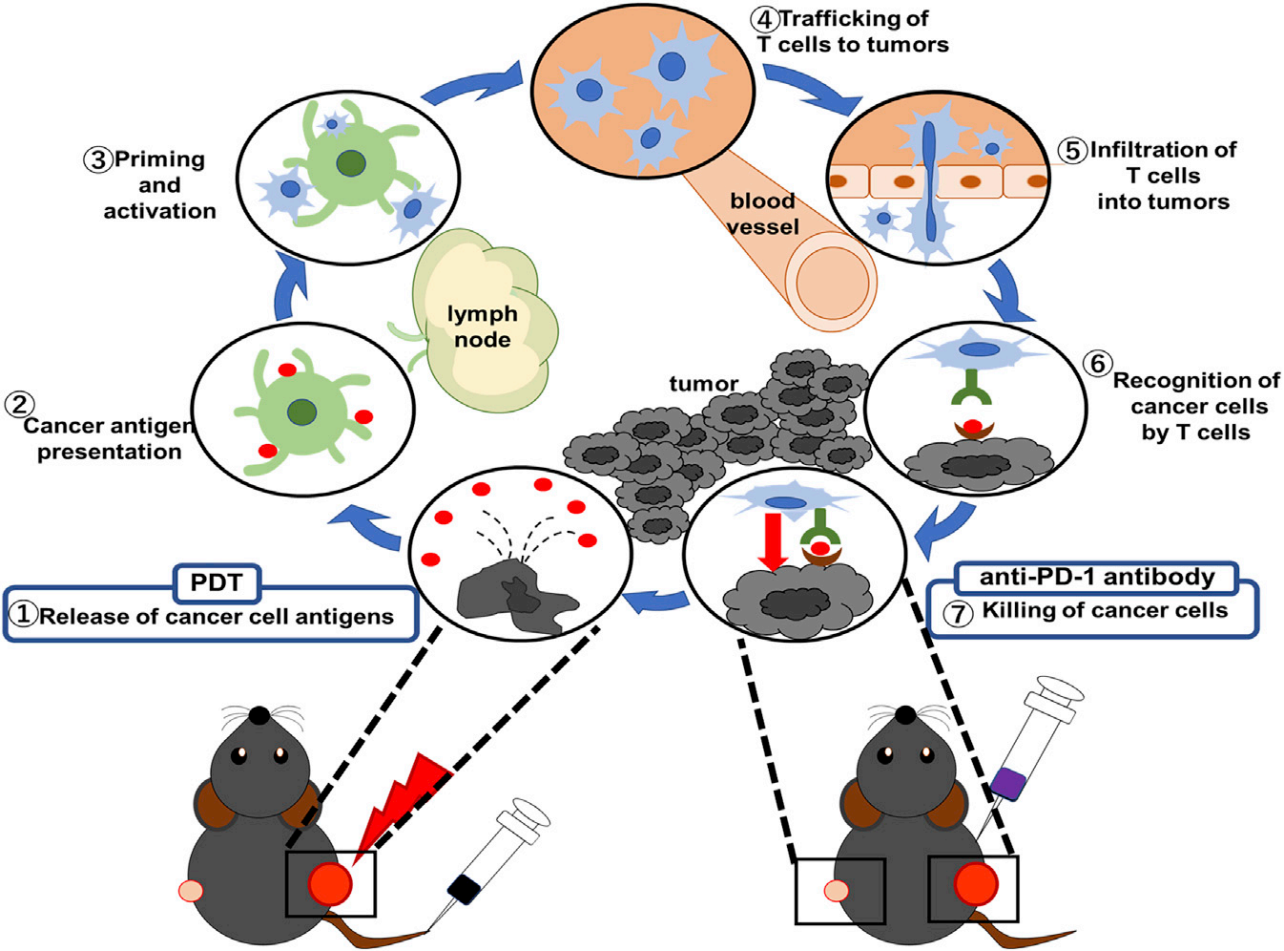
The cancer immunity cycle and roles of PDT and immune checkpoint inhibitors Schema of the cancer immunity cycle. Tumor cell killing by TS-PDT promotes the release of neoantigens and induction of DAMPs (step 1), and anti-PD-1/PD-L1 pathway blockage induces killing of cancer cells by T cells (step 7). Following these steps, the synergistic effect is induced. This figure is a modification of the figure by Chen et al*.*^25^

Based on these results, we considered the synergism of TS-PDT and anti-PD-1 antibody *in vivo* using an immunocompetent mouse tumor model. The combination of TS-PDT and anti-PD-1 antibody inhibited tumor growth compared with other single treatment or non-treatment groups. It is notable that this result was obtained not only from the irradiated side, but also from the non-irradiated side. It is possible that the colon cancer flank tumors established in syngeneic immunocompetent mice did not reflect the real tumor microenvironment, which is a limitation of this experiment. We considered that TS-PDT induces an abscopal tumor-specific immune response in both irradiated and non-irradiated tumors, which is potentiated by PD-1/PD-L1 pathway blockade. The expression levels of CD8^+^ cells and CD4^+^ cells were increased in the combination therapy group. Monotherapy of TS-PDT or anti-PD-1 antibody could promote CTLs and induce anti-cancer effects, but the combination therapy could activate more CTLs compared with each monotherapy. IFN-γ, perforin 1, and granzyme B expression tended to increase in the combination therapy groups. These molecules reflect the activation of CTLs. In our opinion, the synergism of TS-PDT and anti-PD-1 antibody can be elucidated by the cancer immunity cycle (Figure 7). At first, neoantigens are released from dying cells and captured by DCs (step 1). Next, DCs present the captured antigens to T cells (step 2), resulting in the priming and activation of effector T cell responses (step 3). Finally, the activated effector T cells traffic to (step 4) and infiltrate the tumor (step 5); furthermore, they recognize and bind to specific cancer cells (step 6) and kill their target cancer cells (step 7). In this cycle, tumor cell killing by TS-PDT promotes the release of neoantigens and induction of DAMPs (step 1), and anti-PD-1/PD-L1 pathway blockage induces killing of cancer cells by T cells (step 7). We consider that stimulation at these two different points in this cycle can accelerate the rotation of cancer immunity.

In conclusion, TS-PDT not only induced a direct killing effect on tumor cells, but also enhanced the anti-tumor immunity, and more, exhibited synergism with the anti-PD-1 antibody. Therefore, we believe that the combination of TS-PDT and anti-PD-1 antibody may be used to develop potential anti-tumor therapeutic strategies.

## Materials and methods

No patient-derived material was used in this study and thus there is no patient-specific ethical approval to report. The protocols for all animal studies are also used by Nagoya City University Center for Experimental Animal Science, and the mice were treated according to the guidelines of Nagoya City University for animal experiments under permit 19-024. All experiments involving animals were performed under anesthesia, and all efforts were made to minimize their suffering.

### Chemical reagents

TS (mono-l-aspartyl chlorin 6; Laserphyrin) was obtained from Meiji Seika (Tokyo, Japan). Mouse anti-PD-1 antibody (4H2) was provided by Ono Pharmaceutical (Osaka, Japan).

### Cell culture

The esophageal cancer cell line KYSE30 (94072011; European Collection of Authenticated Cell Cultures, Salisbury, UK) was cultured in a 1:1 mixture of Ham’s F12 medium (Wako Pure Chemical Industries, Tokyo, Japan) and RPMI 1640 medium (Wako Pure Chemical Industries) supplemented with 10% heat-inactivated fetal bovine serum (FBS; Biosera, Nuaillé, France) and 1% ampicillin and streptomycin (Thermo Fisher Scientific, Waltham, MA, USA). The human colon cancer cell line HCT116 (CCL-247; American Type Culture Collection, Manassas, VA, USA) was cultured in RPMI 1640 medium (Wako Pure Chemical Industries) supplemented with 10% FBS and 1% ampicillin and streptomycin (Thermo Fisher Scientific). Murine colon adenocarcinoma cell line MC38 (ENH204-FP; Kerafast, Boston, MA, USA) was cultured in Dulbecco’s modified Eagle medium with high glucose (Wako Pure Chemical Industries) supplemented with 10% FBS, 1 mM sodium pyruvate (Thermo Fisher Scientific), 100 μM non-essential amino acids (Thermo Fisher Scientific), 50 μg/mL gentamicin (Wako Pure Chemical Industries), 10 μM 4-(2-hydroxyethyl)-1-piperazineëthanesulfonic acid (Thermo Fisher Scientific), and 1% penicillin-streptomycin-amphotericin B (Wako Pure Chemical Industries). Cells were cultured in an atmosphere of 5% carbon dioxide (CO_2_) at 37°C. All experiments using cells in this study were performed for fewer than 20 passages after thawing.

### Flow cytometric analysis for TS accumulation

Cells were seeded in 6-cm culture dishes and incubated for 24 h. Following this, the medium was replaced with fresh medium supplemented with TS and added to the dishes for 0, 30, 60, 120, 240, and 360 min to evaluate the accumulation of TS in cells. TS was added to the culture medium at a final concentration of 20, 60, and 25 μmol/L for KYSE30, HCT116, and MC38, respectively. After the cells were washed with PBS, they were removed for analysis using a flow cytometer with excitation and emission at 488 and 690 nm. All flow cytometric examinations were performed in triplicate on the FACSCanto II (BD Biosciences, Ann Arbor, MI, USA), and 10,000 events were counted and analyzed using the FlowJo software (BD Biosciences).

### Detection of subcellular localization of TS

Cells were seeded onto eight-well glass slides (Nalge Nunc International, Rochester, NY, USA) and incubated for 24 h. Subsequently, TS (20 μmol/L) was added to the culture medium and the cells were incubated further for 2 h for staining with organelle-specific fluorescent probes. Lysosomes were stained with 0.1 μmol/L LysoTracker green (Thermo Fisher Scientific) at 37°C in 5% CO_2_ for 30 min, mitochondria with 0.1 μmol/L MitoTracker Green FM (Thermo Fisher Scientific) at 37°C in 5% CO_2_ for 10 min, Golgi with 5 μmol/L NBD C6-ceramide (Thermo Fisher Scientific) at 4°C on ice for 30 min, and endoplasmic reticulum with 0.1 μmol/L ER-Tracker green (Thermo Fisher Scientific) at 37°C in 5% CO_2_ for 30 min. After incubation under each condition, the culture media were replaced with fresh media to remove the free dyes. The stained cells were examined using a confocal laser microscope (FV3000; Olympus, Tokyo, Japan) and the CellSens imaging software (Olympus). Band-pass emission filters of 505–540 and 488 nm for organelle-specific fluorescent probes and 570–670 and 561 nm for TS were used. The fluorescence intensity profiles of TS and the organelle probe were examined using confocal microscopy. Fluorescence intensity profiles were examined via confocal imaging, and quantitative analysis was performed using the CellSens imaging software. Data from three independent experiments are presented as the mean ± standard error (SE).

### PDT *in vitro*

Cells were incubated with varying concentrations of TS (depending on each experiment). The cells were washed with PBS to remove the free TS and irradiated using a light-emitting diode system (OptoCode, Tokyo, Japan) at a light energy dose of 16 J/cm^2^ (irradiance: 30.8 mW/cm^2^ × 520 s) with a wavelength of 660 nm.

### Cell viability assay

Cell viability was assessed using the WST-8 cell proliferation assay. Cells were seeded in 96-well culture plates and incubated for 24 h. The cells were then subjected to *in vitro* PDT with various doses of TS and incubated further for 2 h. The cells were then irradiated and incubated with the culture medium for a further 24 h, followed by incubation with the Cell Counting Kit-8 (Dojindo, Kumamoto, Japan) for 2 h, and absorbance was measured at 450 nm using a microplate reader (SPECTRA MAX340; Molecular Devices, San Jose, CA, USA). Cell viability was expressed as the percentage of untreated control cells. IC_50_ was calculated from the survival curve. Data are expressed as the mean ± SE from three independent experiments.

### Flow cytometric analysis of the apoptosis/necrosis of cells stained with annexin V-FITC and PI

Cells were seeded and incubated for 24 h. Following this, the medium was replaced with fresh medium supplemented with TS. TS was added to the culture medium at a final concentration of 40 μmol/L for KYSE30, 60 μmol/L for HCT116, and 25 μmol/L for MC38. The cells were incubated for 24 h, and *in vitro* TS-PDT was administered. After exposure to irradiation, the cells were cultured for 6 or 24 h. After each culture medium was collected and washed with PBS, the cells were removed. The removed cells and the corresponding culture media were centrifuged to obtain cell pellets. Each cell pellet was stained with the MEBCYTO Apoptosis kit (Medical & Biological Laboratories, Tokyo, Japan). All flow cytometric examinations were performed in triplicate on the FACSCanto II and analyzed using the FlowJo software.

### Detection of activated caspase-3 by flow cytometer

Cells were seeded and incubated for 24 h. Following this, the medium was replaced with fresh medium supplemented with TS. TS was added to the culture medium at a final concentration of 40 μmol/L for KYSE30, 60 μmol/L for HCT116, and 25 μmol/L for MC38. The cells were incubated for 24 h, and *in vitro* PDT was administered. After exposure to irradiation, the cells were cultured for 6 or 24 h and the culture medium was collected. After the cells were washed with PBS, they were removed. The removed cells and the corresponding culture media were centrifuged to obtain cell pellets. Each cell pellet was stained with a PE Active Caspase-3 Apoptosis Kit (BD Biosciences, Ann Arbor, MI, USA). All flow cytometric examinations were performed in triplicate on the FACSCanto II and analyzed using the FlowJo software.

### Western blotting analysis of LC3

Cells were seeded and incubated for 24 h. Following this, the medium was replaced with fresh medium supplemented with TS. TS was added to the culture medium at a final concentration of 2.5 μmol/L for KYSE30, 10 μmol/L for HCT116, and 5 μmol/L for MC38. Twenty-four hours after TS-PDT, the intracellular proteins were extracted from the cells. The protein samples were then separated on Mini-PROTEAN TGX gels (Bio-Rad Laboratories, Hercules, CA, USA) and the protein bands were transferred onto a nitrocellulose membrane (Schleicher and Schuell BioScience, Dassel, Germany). The membrane was incubated with the primary anti-LC3 antibody (1:1,000 dilution; 12741; Cell Signaling Technology, Danvers, MA, USA) after blocking. Thereafter, the membrane was washed extensively and incubated with the secondary anti-rabbit IgG horseradish peroxidase (HRP)-linked antibody (1:5,000 dilution; 7074; Cell Signaling Technology). The signals were quantified using the ECL Plus Western Blotting Detection System (GE Healthcare, Chicago, IL, USA), Image Quant LAS 4000 Mini System (GE Healthcare), and ImageJ software. The membranes were also probed with anti-β-actin antibody (1:1,000 dilution; 010-27841; FUJIFILM, Tokyo, Japan) and anti-mouse IgG HRP-linked antibody (1:5,000 dilution; 7076; Cell Signaling Technology) as internal controls. Internal controls were probed using the same blot used for the experimental samples. The results presented as images were from one representative experiment of four experiments, and the bar graph indicates the mean ± SE of four independent experiments.

### Autophagy quantitative analysis using DAP green dye

The cells were seeded in 96-well culture plates and incubated for 24 h. The cells were then incubated with TS and DAP green (Dojindo) for 2 h. The final concentration of TS was 5 μmol/L for all cell lines and that of DAP green was 0.2, 0.8, and 0.4 μmol/L for KYSE30, HCT116, and MC38, respectively. Cells were washed twice with PBS, treated with *in vitro* PDT, and incubated with the culture medium for a further 24 h. Fluorescence was then observed using a microplate reader with excitation/emission at 450/535 nm (Gemini EM microplate reader; Molecular Devices). Data are presented as the mean ± SE (n = 6–7).

### Flow cytometric analysis for the detection of cell-surface ICD markers

Cells were seeded and incubated for 24 h. Following this, the medium was replaced with fresh medium supplemented with TS. TS was added to the culture medium at a final concentration of 20 μmol/L for KYSE30, 60 μmol/L for HCT116, and 25 μmol/L for MC38. The cells were incubated further for 24 h, washed once, and immersed in PBS, and *in vitro* PDT was administered. The cells were further incubated for 15 min and the cell-surface expression levels of CRT (1:350 dilution; ab2907; Abcam, Cambridge, UK) and HSP90 (1:1,000 dilution; ab203126; Abcam) were analyzed by flow cytometry. The secondary antibody used was Alexa Fluor 488 goat anti-rabbit IgG (Thermo Fisher Scientific). All flow cytometry examinations were performed in triplicate on the FACSCanto II counting 10,000 events and analyzed using the FlowJo software.

### Immunocytochemistry for the detection of cell-surface ICD markers

Cells were seeded onto eight8-well glass slides (Thermo Fisher Scientific) and incubated for 24 h. Following this, the medium was replaced with fresh medium supplemented with TS. TS was added to the culture medium at a final concentration of 20 μmol/L for KYSE30, 60 μmol/L for HCT116, and 25 μmol/L for MC38. Cells were incubated with TS for 2 h, and *in vitro* PDT was administered. The cells were fixed with ethanol and acetone for 2 h after *in vitro* PDT. Primary antibodies against CRT (1:1,000 dilution; ab2907; Abcam) or HMGB1 (1:250 dilution; ab79823; Abcam) were used. The secondary antibody used was Alexa Fluor 488 goat anti-rabbit IgG (Thermo Fisher Scientific). All sections were counterstained with DAPI (Kirkegaard and Perry Laboratories, Gaithersburg, MD, USA). Images were obtained using a confocal laser microscope (FV3000) and the CellSens imaging software (Olympus). Band-pass emission filters (405 and 488 nm) were used.

### Measurement of the release of HMGB1 by enzyme-linked immunosorbent assay (ELISA)

Cells were seeded and incubated for 24 h. Following this, the medium was replaced with fresh medium supplemented with TS. TS was added to the culture medium at a final concentration of 20 μmol/L for KYSE30, 60 μmol/L for HCT116, and 25 μmol/L for MC38. The cells were incubated further for 24 h, washed once with PBS, immersed in 1 mL/well RPMI 1640 without phenol red (Wako Pure Chemical Industries), and then *in vitro* PDT was administered. Following this, the cells were incubated for 0, 5, 15, 30, 60, 120, 240, and 360 min before analysis. The medium was collected and HMGB1 concentration was measured using the HMGB1 ELISA Kit II (Shino-Test, Kanagawa, Japan). The absorbance was measured at 450 nm using a microplate reader (SPECTRA MAX340), and the HMGB1 concentration in each sample was calculated based on a standard curve. Data are presented as the mean ± SE (n = 3).

### Measurement of the release of ATP by ELISA

Cells were seeded and incubated for 24 h. Following this, the medium was replaced with fresh medium supplemented with TS. TS was added to the culture medium at a final concentration of 20 μmol/L for KYSE30, 60 μmol/L for HCT116, and 25 μmol/L for MC38. The cells were incubated further for 24 h, washed with PBS, and immersed in 1 mL/well RPMI 1640 without phenol red, and then *in vitro* PDT was administered. Following this, the cells were incubated for 0, 5, 15, 30, 60, 120, 240, and 360 min before analysis. ATP concentration was measured using the ENLITEN ATP Assay System bioluminescence detection kit for ATP (Promega, Madison, WI, USA). The ATP concentration in each sample was calculated based on a standard curve. Data are presented as the mean ± SE (n = 3).

### Real-time potency assay

Human PBMCs were obtained from iQ Bioscience (Berkeley, CA, USA). SEB was obtained from Toxin Technology (Sarasota, CA, USA). The xCELLigence RTCA DP (Agilent Technologies, Santa Clara, CA, USA) was used for all impedance experiments. First, 100 μL of RPMI 1640 culture medium was added to each well of 16-well E-plates (Agilent Technologies), and the background impedance was measured. Dissociated adherent target cells (HCT116) were seeded at a density of 3 × 10^4^ cells/well in a volume of 100 μL and allowed to passively adhere to the electrode surface. After seeding, the E-plate was kept at ambient temperature inside a laminar flow hood for 30 min and then transferred to the RTCA DP instrument inside a cell culture incubator. Data recording was initiated immediately at 15 min intervals for the entire duration of the experiment. Thereafter, the target cells were incubated for 12 h. Human PBMCs were stimulated with SEB to increase the expression of PD-1 on the surface of immune cells for 48 h before addition to HCT116. We considered four treatment groups as follows: (1) anti-PD-1 antibody treatment: 100 μL of medium in each well was aspirated and replaced with 200 μL of medium containing 10 nmol/L anti-human PD-1 monoclonal antibody (eBioJ105 [J105]; Thermo Fisher Science) and 6.5 × 10^4^ cells/well of PBMCs. (2) TS-PDT treatment: the medium in each well was replaced with 100 μL medium containing 4 μmol/L TS and incubated for 2 h. Then, the medium was washed with PBS and replaced with 100 μL PBS, and *in vitro* PDT was administered. After exposure to irradiation, the cells were cultured in 200 μL medium containing 6.5 × 10^4^ cells/well PBMCs. (3) Combination treatment: the addition of medium containing 10 nmol/L anti-PD-1 antibody and 6.5 × 10^4^ cells/well of PBMCs was followed by the PDT treatment procedure as mentioned above. (4) Untreated group, the culture medium was changed to 200 μL medium containing 6.5 × 10^4^ cells/well PBMCs at the time of treatment with other groups. Changes in impedance were reported as the normalized cell indices. Combination index was calculated by CompuSyn software. Quantitative analysis was performed using the RTCA software 2.0 (Agilent Technologies). Data are presented as the mean (n = 6).

### Animal models and inhibition of tumor growth

All our experiments were performed using female mice for the animal models (C57BL/6), 6–8 weeks of age, 18–22 g body weight, purchased from the Shizuoka Laboratory Animal Center (Shizuoka, Japan). Mice were kept under pathogen-free conditions with controlled temperature and humidity, 12 h light/dark cycle conditions, and fed a sterilized pellet diet with water *ad libitum*. Before any interventions were started, all mice were acclimatized for at least 2 weeks in the animal facility. Syngeneic mouse models of flank tumors were established by subcutaneously implanting 5 × 10^5^ MC38 cells in 100 μL of medium into the right flank and 1.25 × 10^5^ MC38 cells in 25 μL of medium into the left side of the mice (day 0). For anti-PD-1 antibody monotherapy, mice were intraperitoneally administrated 2.5 mg/kg anti-PD-1 antibody (4H2) 6 days after tumor inoculation (day 6). The therapy was repeated five times every 3 days. For TS-PDT monotherapy, TS was administered to mice via the tail vein at a dose of 3.125 μmol/kg. Two hours after administration, the tumors were irradiated using a 664 nm red laser (OK Fiber Technology, Kyoto, Japan) at a dose of 2 J/cm^2^ (intensity 150 mW/cm^2^) applied to the skin directly above the tumors. PDT was performed only once on day 6. For combination therapy, mice were intraperitoneally administered 2.5 mg/kg anti-PD-1 antibody and then treated with TS-PDT following the same method mentioned above. In every group, tumor growth was monitored once every 3 days by measuring the tumor volume with Vernier calipers. Simultaneously, the health of the mice was monitored. Mice with a tumor size of 1,000 mm^3^ were designated to be euthanized. None of the mice were found to be unhealthy or dead throughout this study. Cervical dislocation was used for euthanasia. Mice were anesthetized via an intraperitoneal injection of ketamine (100 mg/kg) and xylazine (10 mg/kg) reconstituted in physiological saline solution. The data were calculated as the relative tumor volume (the respective tumor on day 6) and presented as the mean ± SE (n = 8–9).

### Immunohistochemistry

On day 15, the tumors were immediately excised from xenograft models, fixed in formalin, and embedded in paraffin blocks. The block specimens were then sectioned (4 μm) and stained with Bond Max (Leica Microsystems, Wetzlar, Germany). Anti-CD4 antibody (1:500 dilution; ab183685; Abcam), anti-CD8 antibody (1:500 dilution; 98941; Cell Signaling Technology), anti-granzyme B antibody (1:100 dilution; ab4059; Abcam), and anti-PD-L1 antibody (2 μg/mL; ab205921; Abcam) were used for MC38 tumors after *in vivo* examination. All immunohistochemical staining was performed using a standard immunoperoxidase technique (Histofine SAB-PO Kit; Nichirei, Tokyo, Japan). Fifteen random fields from each sample were captured under a microscope (Nikon ECLIPSE 80i; Nikon, Tokyo, Japan) at a magnification of 400×, and the areas stained with antibodies were counted. Data from each group (n = 5) are expressed as the mean ± SE.

### Quantitative reverse transcription (qRT)-PCR analysis

On day 15, the tumors were immediately excised from the syngeneic mouse tumor models. IFN-γ, perforin 1, granzyme B, and glyceraldehyde 3-phosphate dehydrogenase (GAPDH) mRNA expression levels in MC38 tumors were measured by qRT-PCR. GADPH was chosen as an endogenous control to normalize the expression data. mRNA was reverse transcribed into cDNA using a high-capacity cDNA reverse transcription kit according to the manufacturer’s instructions. PrimeTime qPCR assays for IFN-γ (Mm.PT.58.41769240), perforin 1 (Mm.PT.58.41904164), and granzyme B (Mm.PT.58.42155916) were purchased from Integrated DNA Technologies (Coralville, IA, USA), and mouse GAPDH (NM_008084.2) was purchased from Thermo Fisher Scientific. qRT-PCR analyses were performed in triplicate using an ABI 7500 Fast real-time PCR system (Thermo Fisher Scientific) according to the supplier’s recommendations. All data are presented as the fold change of the internal control GAPDH. Data from each group (n = 5) are expressed as the mean ± SE.

### Statistical analysis

Descriptive statistics was used and the samples were analyzed using the Prism software v.6.0 (GraphPad Software, San Diego, CA, USA). Statistical significance was determined by Student’s t test between two groups or Holm-Sidak’s multiple comparisons test. The log-rank test was performed to compare tumor volume suppression curves (∗p < 0.05 and ∗∗p < 0.01).

## Data Availability

The datasets generated and/or analyzed during the current study are available from the corresponding author upon reasonable request.

## Supplemental information

Document S1. Figures S1–S4, Table S1, and Supplemental materials and methods

Document S2. Article plus supplemental information
