# Supplementary material for: Anti-tumor immunity enhancement by photodynamic therapy with talaporfin sodium and anti-programmed death 1 antibody
Source: Mol Ther Oncolytics. 2023 Jan 2;28:118–31. doi: 10.1016/j.omto.2022.12.009 (PMC9867957; doi:10.1016/j.omto.2022.12.009)
Supplement: Document S1. Figures S1–S4, Table S1, and Supplemental materials and methods [file mmc1.pdf]

OMTO, Volume *28*

Supplemental information

Anti-tumor immunity enhancement

by photodynamic therapy with talaporfin sodium

and anti-programmed death 1 antibody

Makiko Sasaki, Mamoru Tanaka, Yuki Kojima, Hirotada Nishie, Takaya Shimura, Eiji Kubota, and Hiromi Kataoka

Supporting Information

Supporting Materials and Methods

Real-time potency assay

Human peripheral blood mononuclear cells (PBMCs) were obtained from iQ Bioscience (Berkeley, CA, USA). Staphylococcal enterotoxin B (SEB) was obtained from Toxin Technology, Inc. (Sarasota, CA, USA). The xCELLigence RTCA DP (Agilent Technologies, Inc., Santa Clara, California, USA) was used for all impedance experiments. First, 100 μL of RPMI-1640 culture medium was added to each well of 16-well E-Plates (Agilent Technologies), and the background impedance was measured. Dissociated adherent target cells (HCT116) were seeded at a density of 3×10^4 cells/well in a volume of 100 μL and allowed to passively adhere to the electrode surface. After seeding, the E-Plate was kept at ambient temperature inside a laminar flow hood for 30 min and then transferred to the RTCA DP instrument inside a cell culture incubator. Data recording was initiated immediately at 15 min intervals for the entire duration of the experiment.

(A) Thereafter, the target cells were incubated for 20 h and several concentrations of TS (0, 4, 8μM) were added. Following this, *in vitro* PDT was performed and cell proliferation curves were obtained.

(B) Thereafter, the target cells were incubated for 20 h and human PBMCs were stimulated with SEB to increase the expression of PD-1 on the surface of immune cells for 48 h before addition to HCT116. 100 μL of medium in each well was aspirated and replaced with 200 μL of medium containing 0, 5, 10 and 20 nmol/L of anti-human PD-1 monoclonal antibody (eBioJ105 (J105); Thermo Fisher Science) and 6.5×10^4 cells/well of PBMCs.

Changes in impedance were reported as the normalized cell indices. Combination Index was calculated by CompuSyn software. Quantitative analysis was performed using the RTCA Software 2.0 (Agilent Technologies). Data are presented as the mean (n = 6).

Immunohistochemistry

On day 15, the tumors were immediately excised from xenograft models, fixed in formalin, and embedded in paraffin blocks. The block specimens were then sectioned (4 μm) and

stained with Bond Max (Leica Microsystems, Wetzlar, Germany). CRT (1:400 dilution; bs-5913R; Bioss Inc.,Woburn, MA, USA) and HMGB1 (1:400 dilution; ab79823; Abcam) were used for MC38 tumors after *in vivo* examination. All immunohistochemical staining were performed using a standard immunoperoxidase technique (Histofine SAB-PO Kit). Fifteen random fields from each sample were captured under a microscope (Nikon ECLIPSE 80i; Nikon Corporation) at a magnification of 400× and the areas stained with antibodies were counted. Data from each group (n = 5) are expressed as the mean ± SE.

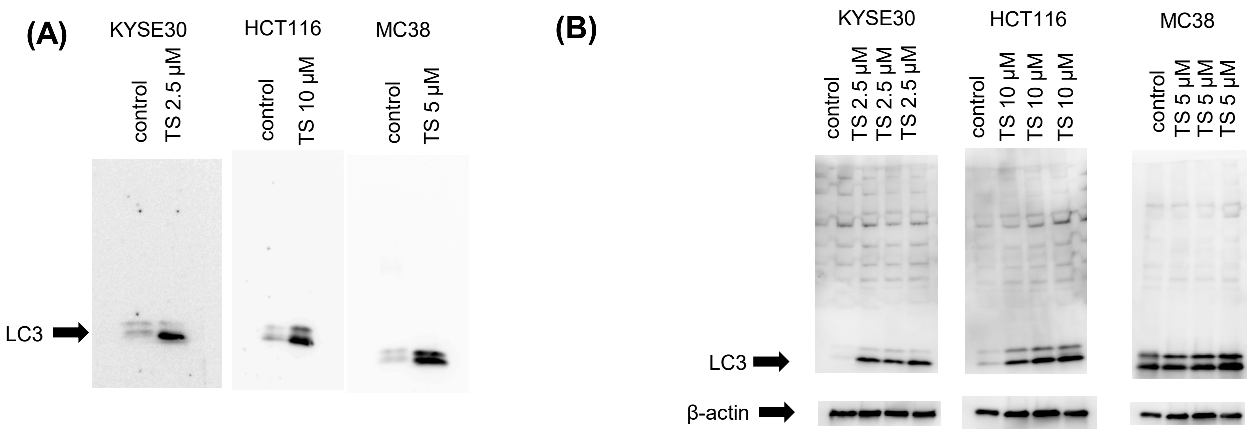

Figure S1 Supplemental data for western blotting.
(A) Whole western blots (uncropped blots) of Figure 2D. (B) Remaining three samples used to calculate relative expression of LC3 normalized to β-actin. Whole western blots and cropper blots are shown.

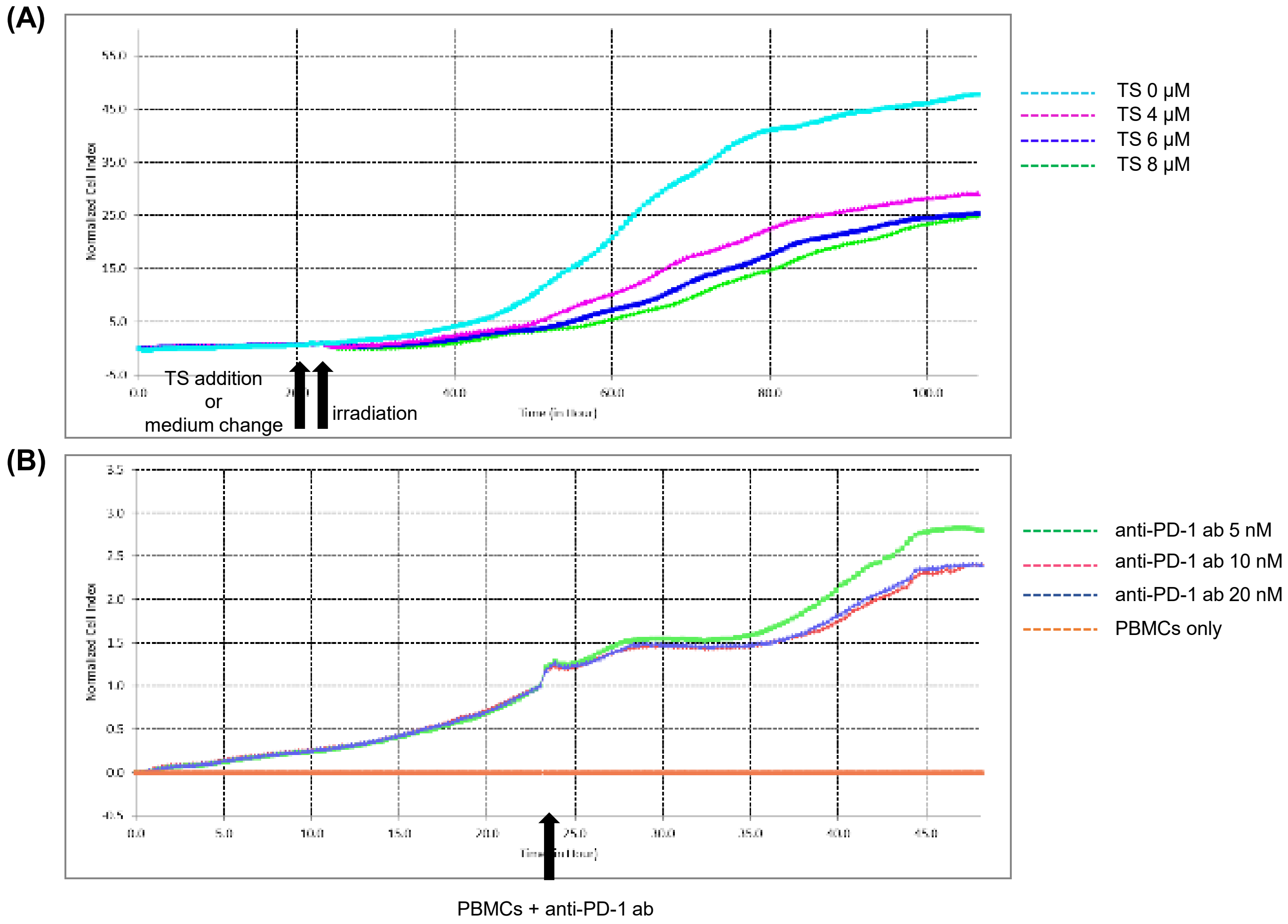

Figure S2 Supplemental data for Figure 4.
(A) Proliferation assay by talaporfin sodium-photodynamic therapy (TS-PDT). HCT116 cells were seeded at a density of 3×10^4 cells/well and several concentrations of TS were added. Following this, *in vitro* PDT was performed and cell proliferation curves were obtained. (B) Proliferation assay by anti-programmed death 1 (anti-PD-1) antibody. HCT116 cells were seeded at a density of 3×10^4 cells/well, and 6.5×10^4 cells/well of peripheral blood mononuclear cells (PBMCs) and several concentrations of anti-PD-1 antibody were added. Following this, cell proliferation curves were obtained. Wells with only PBMCs (HCT116 cells were not seeded) showed that floating PBMCs did not affect the impedance experiments.

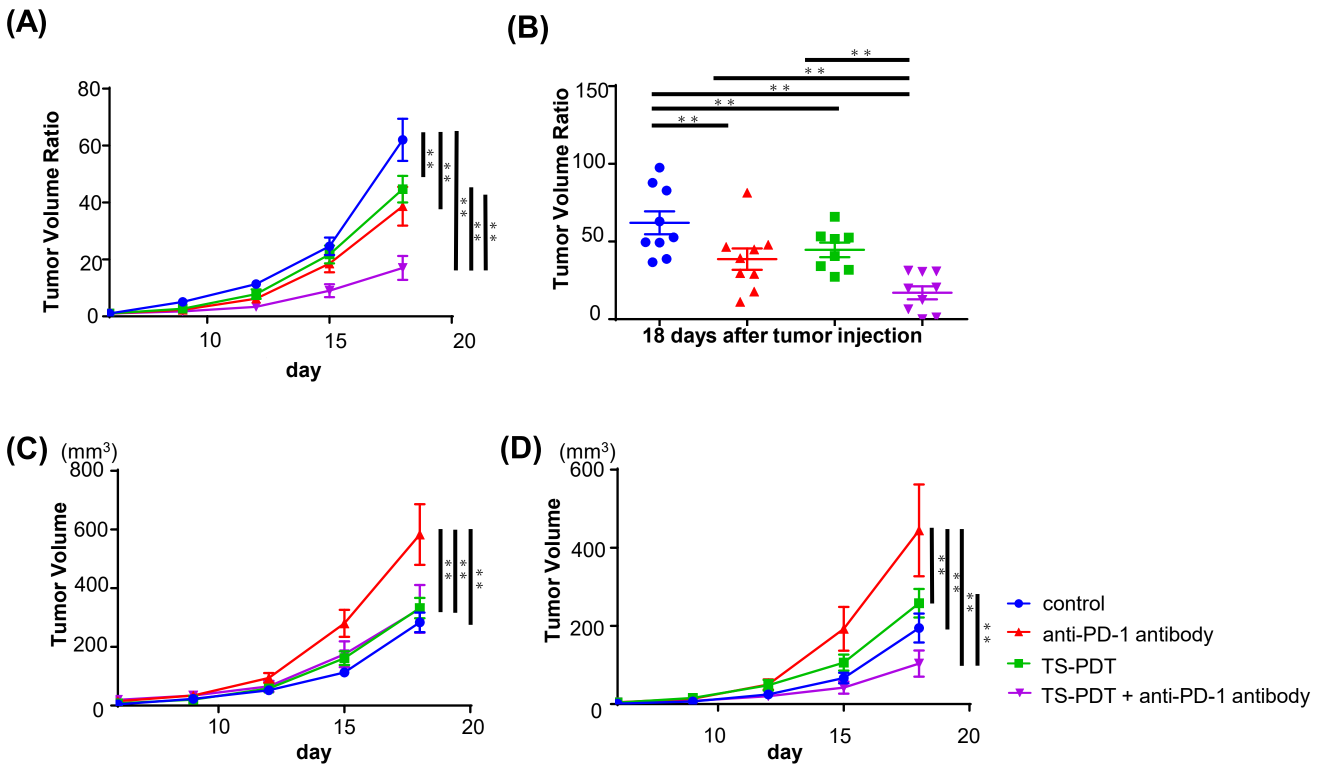

Figure S3. Tumor volume suppression in irradiated side by TS-PDT with anti-PD-1 antibody *in vivo* and tumor volume curves in both side. (A) Tumor volume ratio of irradiated–side plotted against the number of days. (B) Effect of combination treatment with TS-PDT with anti-PD-1 antibody on day 18 (end-point) after tumor implantation of irradiated-side. Tumor volume curves in irradiated side and non-irradiated side is shown in (C) and in (D) respectively. Values are expressed as the mean ± SE (n = 8-9). **P < 0.01 (two-way ANOVA with Holm-Sidak's multiple comparisons test).

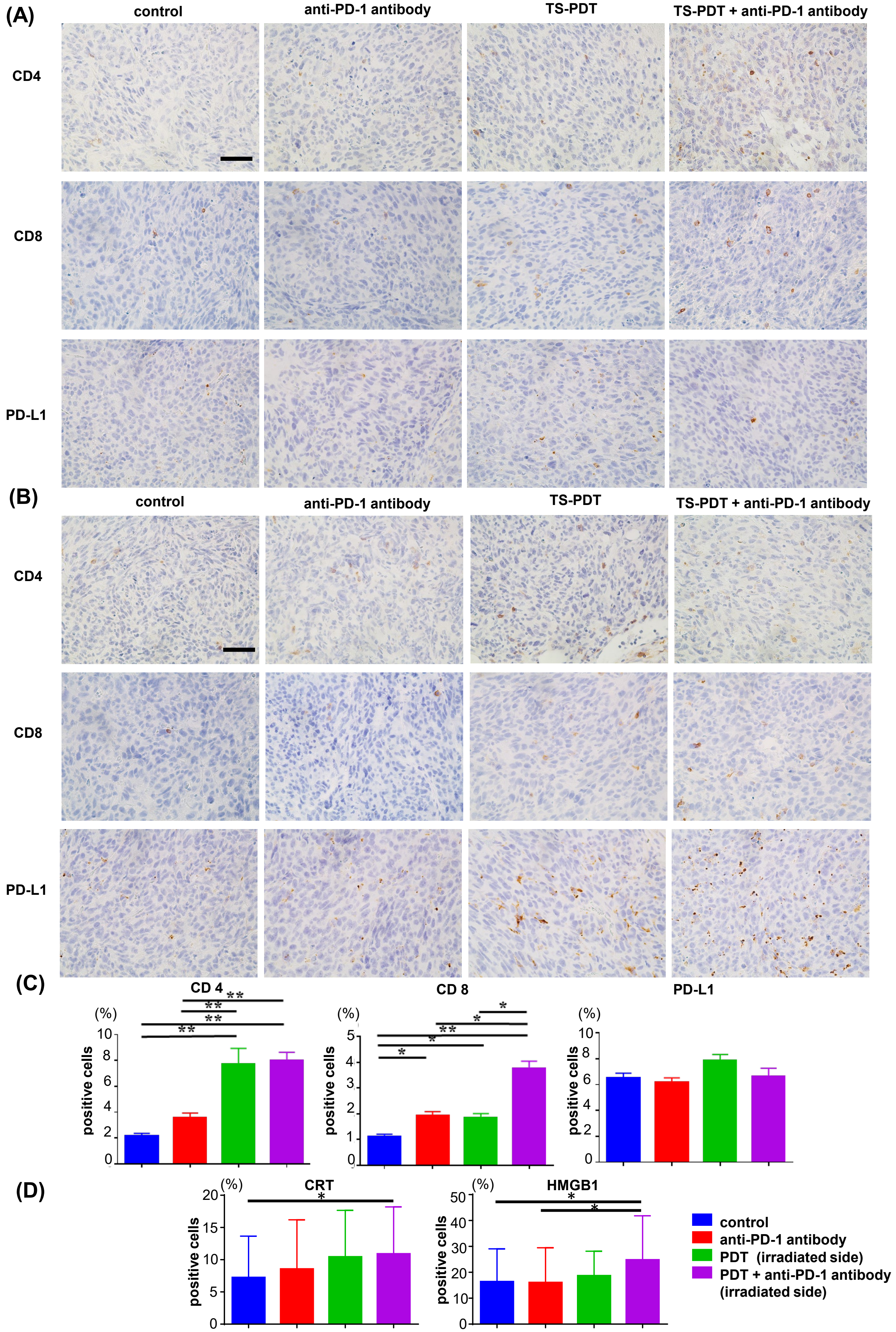

Figure S4. Pathological expression levels of cluster of differentiation (CD)-4, CD8, programmed death-ligand 1 (PD-L1) and damage-associated molecular patterns (DAMPs) *in vivo*.
(A) Non-irradiated side belongs to Figure 6. (B) and (C) Images of irradiated side tumors and values of expression levels. (original magnification, 400 x ; scale bar, 500 μm) (D) The expression levels of (calreticulin (CRT) and high mobility group box 1 (HMGB1)) in tumors. Values are expressed as the mean ± SE (n = 5). *P < 0.05, and **P < 0.01 (Holm-Sidak's multiple comparisons test).

Table S1. The previous reports list of PDT-triggered ICD plus immune checkpoint inhibitors.

Photosensitizer	Immune Checkpoint Inhibitors	Ref
HS201	anti-PD-L1 antibody	35
ICG	anti-PD-L1 antibody<br>anti-CTLA4 antibody	36
porphyrin-based PLZ4-nanoparticles	anti-PD-1 antibody	37
ICG loaded micelles with hyaluronic acid corona	anti-PD-1 antibody	38
verteporfin	anti-CTLA-4 antibody	39
Oxa(IV)@ZnPc@M	anti-PD-L1 antibody	40
WST11	anti-PD-1 antibody	41
mTHPC@VeC/T-RGD nanoparticles	anti-PD-L1 antibody	42
Ce6-embedded nano-photosensitizer	anti-PD-L1 antibody	43
TPE-IQ-2O	BMS202 (PD-1/PD-L1 inhibitor 2)	44
multifunctional covalent organic framework -based nanocomposite	anti-PD-L1 antibody	45
liposomal form of benzoporphyrin derivative monoacid ring	anti-CTLA-4 antibody	46
OR141	anti-CTLA-4 antibody	47
FA-CuS/DTX@PEI-PpIX-CpG nanocomposites	anti-PD-L1 antibody	48
Bristol-Myers Squibb nanoparticles	anti-PD-L1 antibody	49
oly(ethylene glycol)-modified nanoscale TBP-MOF	anti-PD-1 antibody	50
nanoscale coordination polymer core-shell nanoparticles carry oxaliplatin in the core conjugated pyropheophorbide-lipid	anti-PD-L1 antibody	16
integrin αvβ6-specific near-infrared phthalocyanine dye-labeled agent	anti-PD-1 antibody	51
